# Association of smoking behavior among Chinese expectant fathers and smoking abstinence after their partner becomes pregnant: a cross-sectional study

**DOI:** 10.1186/s12884-020-03148-8

**Published:** 2020-08-05

**Authors:** Wei Xia, William Ho Cheung Li, Wenzhi Cai, Peige Song, Laurie Long Kwan Ho, Ankie Tan Cheung, Yuan Hui Luo, Chunxian Zeng, Li He, Chao Gao, Ka Yan Ho

**Affiliations:** 1 School of Nursing, The University of Hong Kong, 4/F, William M. W. Mong Block, 21 Sassoon Road,Pokfulam, Hong Kong SAR, China; 2 Shenzhen hospital, Southern Medical University, Shenzhen, Guangdong China; 3 School of Public Health, Zhejiang University School of Medicine, Zhejiang University, Hangzhou, Zhejiang China; 4 The People’s Hospital Of Wenshan Prefecture, Wenshan, Yunnan China; 5 The Third People’s Hospital of Yunnan Province, Kunming, Yunnan China; 6 School of Nursing, The Hong Kong Polytechnic University, Hung Ho, Kowloon, Hong Kong SAR, China

**Keywords:** Smoking behavior, Expectant fathers, Maternal and neonatal health, Tobacco abstinence association, Smoking cessation, Lasso regression

## Abstract

**Background:**

Exposure to secondhand smoke (SHS) during pregnancy can cause pregnancy complications and adverse birth outcomes. About 40% of Chinese expectant fathers are smokers and they rarely attempt to quit smoking. There is a paucity of effective smoking cessation services targeting this population. In this study, we assessed the smoking behavior of Chinese expectant fathers and examined its association with smoking abstinence after their partner became pregnant, which is an essential prerequisite for designing effective smoking cessation interventions.

**Methods:**

We conducted a cross-sectional survey in the obstetrics and gynecology clinic of three tertiary hospitals in China. Expectant fathers who smoked at least one cigarette per day for 1 month within the past 12 months were invited to participate in this study. The participants were asked to complete a structured questionnaire that assessed their smoking behaviors before and after their partner became pregnant.

**Results:**

From December 2017 to March 2018, we recruited a total of 466 eligible expectant fathers, among whom 323 (69.3%) were identified as current smokers and 143 (30.7%) were ex-smokers. Using lasso regression, 19 features were selected from among 27 independent variables. The results of the selected multivariable logistic regression model showed that knowledge about the health hazards of smoking among smokers (odds ratio (OR) 1.39; 95% confidence interval (CI) 1.24 to 1.58; *p* < 0.001), knowledge about the health hazards of SHS to pregnant women (OR 1.46; 95% CI 1.09 to 1.97; *p* < 0.001), knowledge about harm to the fetus and newborn (OR 1.58; 95% CI 1.25 to 2.03; *p* < 0.001), and being a first-time expectant father (OR 2.08; 95% CI 1.02 to 3.85; *p* = 0.046) were significantly positively associated with smoking abstinence among expectant fathers after their partner became pregnant. Significantly negative associations were found for severe dysfunctionality in terms of family support (OR 0.48; 95% CI 0.24 to 0.95; *p* = 0.036) and smoking only outside the home (OR 0.81; 95% CI 0.26 to 0.98; *p* < 0.001).

**Conclusions:**

In this study, we identified several factors associated with smoking abstinence among expectant fathers after their partner became pregnant. These findings can guide the development of effective interventions targeting expectant fathers, to help them quit smoking.

## Background

Evidence shows that exposure to secondhand smoke (SHS) during pregnancy can cause pregnancy complications and adverse birth outcomes such as preterm delivery, spontaneous abortion, low birth weight, and even fetal death [1, 2]. According to the National Bureau of Statistics of China, there were approximately 1.4 million pregnant women in China in 2019 [3], and many of them were exposed to SHS. A study involving 2345 pregnant women in five Chinese provinces found that 40% of expectant fathers still smoked during their partner’s pregnancy, and the percentage even increased to 43.79% after their child was born [4]. Another study of 1181 non-smoking Chinese pregnant women found that 75.1% lived with smoking partner and were regularly exposed to SHS [5]. To promote the health of smoking expectant fathers and especially to protect pregnant women and newborns from exposure to SHS, it is vital for health care professionals to implement interventions to help expectant fathers quit smoking.

Previous studies have suggested that expectant or new fathers might be more drawn to smoking cessation interventions that foster their own personal strategies for reducing or quitting smoking [6]. Interventions should be applied for men at this golden opportunity to help them to stop smoking and establish a lifelong healthy lifestyle [7]. A systematic review was conducted to determine the extent of SHS and interventions to reduce SHS among pregnant women in China [8]. The review identified five studies related to SHS prevention interventions to pregnant women in China, and these studies primarily focused on promoting avoidance behaviors of pregnant women and only one additionally included intervention in changing husbands’ smoking behaviors [8]. Nevertheless, these studies showed no significant differences in quit rate between groups and thus, the availability of effective interventions to protect pregnant women from SHS in China is limited [8]. Previous studies conducted in Western countries have evaluated the effectiveness of smoking cessation interventions for expectant fathers [9, 10]. The results of these studies have shown no significant differences between the proposed intervention and control groups. One possible reason for the non-significant findings might be that the proposed interventions were too general and not specific enough to motivate expectant fathers to quit smoking. Hence, a thorough understanding of the smoking behavior of expectant fathers and the factors associated with smoking abstinence after their partner becomes pregnant is an essential prerequisite for the design of appropriate and effective smoking cessation interventions that can help these men to achieve a higher rate of smoking abstinence.

A systematic review of qualitative research examined the barriers and facilitators to smoking cessation experienced by women’s partner during pregnancy and the postpartum period [11]. However, all studies included in the systematic review were conducted in Western countries and small sample sizes were used, which limited the generalizability of the findings to the population of Chinese expectant fathers who smoke. Moreover, tobacco use is an intrinsic and ancient part of Chinese culture; smoking serves a particularly important social function in the forging of connections among individuals [12, 13]. Most Chinese smokers believe that protective biological mechanisms specific to Asian populations make smoking less hazardous than for them than for other populations; many Chinese smokers also believe that it is easy to quit smoking [14]. Influenced by the smoking culture in China, non-smoking women are more tolerant of paternal smoking [15]. Hence, this cultural discrepancy makes it inappropriate to develop interventions targeting Chinese expectant fathers based on the findings from studies conducted in other countries. A review of the literature reveals that to date, no studies have examined the smoking behavior of Chinese smoking expectant fathers. In light of these considerations, the smoking behavior of Chinese smoking expectant fathers and the factors associated with smoking abstinence after their partner becomes pregnant should be explored.

## Methods

### Aim and study design

We conducted a cross-sectional survey to assess the smoking behavior of Chinese smoking expectant fathers and to explore the factors associated with smoking abstinence after their partner becomes pregnant. We targeted expectant fathers who continued to smoke or who quit smoking after their partner became pregnant. Expectant fathers who accompanied their pregnant partner to a prenatal examination at the obstetrics and gynecology clinic of three tertiary hospitals in China were assessed for eligibility to participate in the study. The selected hospitals in this study have the largest obstetrics and gynecology clinics in the study regions. This study was reported following the Strengthening the Reporting of Observational Studies in Epidemiology (STROBE).

### Sampling

Expectant fathers were eligible for this study if they: (1) were aged 18 years or above, (2) smoked at least one cigarette per day for 1 month within the past 12 months, and (3) could read Chinese and communicate in Mandarin. We excluded expectant fathers who were mentally or physically unable to communicate. Those who self-reported as having resumed smoking, or who had an exhaled carbon monoxide level of 4 ppm or above (suggesting smoking within approximately the last 24 h) were identified as current smokers. Those who self-reported as having quit for more than 1 month and whose exhaled carbon monoxide level was less than 4 ppm were identified as ex-smokers.

### Sample size estimation

According to previous literature, 50 of 328 smoking expectant father quit successfully after their partners got pregnant with a quit rate of 15.2% [16]. With a confidence level (CI) of 95% and significance level of 5%, at least 199 participants were needed in this study. The sample size was calculated based on the following formula [17].

$$ \mathrm{N}=\frac{Z_{1-\alpha /2}^2\ast p\left(1-p\right)}{d^2}=\frac{1.96^2\ast 0.152\left(1-0.152\right)}{0.05^2} $$

### Measurements

A demographic questionnaire was administered to collect participants’ background information, including, age, occupation, family income, education level, number of children, activity level, and alcohol use.

A structured standardized questionnaire (Supplementary) was used in this study, which has been well validated in previous studies [18, 19]. The panel comprised an associate professor, an assistant professor from a local university, and a nurse practitioner in gynecology and obstetrics from a local tertiary hospital, all of whom had extensive knowledge of smoking cessation and obstetrics. In the structured standardized questionnaire, participants’ health-related quality of life was assessed using the 12-Item Short-Form Survey (SF-12) as physical health status (PCS) and mental health status (MCS), respectively. The Chinese version of the SF-12 has been tested and shown to have good internal consistency (0.910) and reliability (0.812) [20]. Participants’ nicotine dependency was assessed with the Fagerström Test of Nicotine Dependence (FTND), the coefficient of construct reliability for the Chinese scale has been empirically examined, with a score of 0.74 [21]. Smoking self-efficacy among expectant fathers was assessed using the Smoking Self-Efficacy Questionnaire (SEQ-12). The interclass correlation coefficients of 0.95 and 0.93 for internal stimuli and external stimuli in the Chinese version were obtained, respectively [22]. Family support was evaluated using the Family Adaptation, Partnership, Growth, Affection, and Resolve (Family APGAR) [23]. The Chinese version of the Family APGAR has demonstrated good test–retest reliability (0.91) [24]. This questionnaire also covers the following areas: (1) whether participants have attended prenatal education and received advice regarding smoking cessation from health care professionals at a clinic, (2) smoking and quitting behaviors before and after the partner became pregnant, (3) intention to quit smoking, (4) knowledge about the health hazards of tobacco use to smokers themselves, pregnant women, fetuses, and newborns, (5) attitude toward tobacco use, (6) and risk perception toward smoking using a binary scale (yes or no).

### Data collection

To identify potential participants at each registration center, promotional posters were placed next to the reception desk, highlighting the nature and purpose of the study. All pregnant women were screened for their husbands’ smoking status. Research nurses then invited smoking expectant fathers to participate in the study, after confirming their eligibility. After receiving an explanation of the study details, expectant fathers were informed that their participation was voluntary and without prejudice to them or their partner. Expectant fathers who agreed to participate were asked to provide their written consent and complete the questionnaire, including demographic information. The entire process required about 30 min and took place while expectant fathers were waiting for their partner to undergo a prenatal examination, causing only minimal disturbance to the clinical routine.

### Data analysis

R programming language version 3.1 (The R Project for Statistical Computing, Vienna, Austria) was used to perform all data analyses. Descriptive statistics were used to detail participants’ demographic characteristics and their smoking profile. The frequency and percentage or mean and standard deviation were used to present categorical data and continuous data, respectively.

Lasso regression, a method of feature selection using regularization to minimize prediction error for variables and avoid overfitting, was performed to select variables associated with expectant fathers’ smoking abstinence after their partner became pregnant from all assessed independent variables [25]. The receiver operating characteristic (ROC) curve and Hosmer–Lemeshow test were used to diagnose and evaluate the goodness of fit of the selected model [26, 27]. Multivariable logistic regression using the selected model was then conducted to identify predictors of smoking abstinence in expectant fathers after their partner becomes pregnant.

## Results

Between December 2017 and March 2018, we screened a total 1979 expectant fathers. Of 631 who were eligible, 466 (73.9%) expectant fathers agreed to participate in this study and completed the questionnaire. Of these, 143 (30.7%) were identified as ex-smokers and 323 (69.3%) as current smokers.

Table 1 presents the demographic characteristics of expectant fathers. The mean age of participants was 32.5 (SD = 5.3) years. About 83.3% (388/466) of participants were employed, and 68.9% (321/466) had an education level of college or above. About 58.8% (274/466) of participants were first-time expectant fathers; 25.8% (120/466) of participants lived with other smokers, and the wives of 10.3% (48/466) of participants were also smokers. A total 70.6% (329/466) of participants received satisfactory support from their family whereas 4.5% (21/466) reported severe dysfunctionality in terms of family support. Among participants, 53.4% (249/466) attended prenatal education, and 54.1% (252/466) had received smoking cessation advice from health care professionals.

**Table 1:** Demographic characteristics of the participants (*n* = 466)

	n (%)
Age (range: 20–62), mean (SD)	32.5(5.3)
Employment status	
Employed	388(83.3)
Unemployed/Self-employment	78(16.7)
Education level	
Middle school or less	145(31.1)
College/university or above	321(68.9)
Annual Family income (CNY) ^a^	
¥ 49,999 or below	82(17.6)
¥ 50,000–99,999	119(25.5)
¥ 100,000-199,999	186(39.9)
¥ 200,000or above	79(17.0)
Monthly regular alcohol use	
Yes	333(71.5)
No	133(28.5)
Regular activity at least 1 h/week	
Yes	312(67.0)
No	154(33.0)
Physical health status (SF-12-PCS), mean (SD)	53.2(3.9)
Mental health status (SF-12-MCS), mean (SD)	52.4(6.1)
Stressful event within 30 days	
Yes	209(44.8)
No /Not sure	257(55.2)
First-time expectant father	
Yes	274(58.8)
No	192(41.2)
Living with other smokers	
Yes	120(25.8)
No	346(74.2)
Wife is smoker	
Yes	48(10.3)
No	418(89.7)
Family support function level, by family APAGR	
Satisfactory support from family (8–10)	329(70.6)
Moderate dysfunctionality family support (4–7)	116(24.9)
Severe dysfunctionality family support (0–3)	21(4.5)
Prenatal education attendance	
Yes	249(53.4)
No	217(46.6)
Smoking cessation advice received	
Yes	252(54.1)
No	214(45.9)

Data are n(%) and mean (SD) unless stated otherwise. SF-12 = 12-Item Short-Form Survey. *PCS* Physical Health Status, *MCS* Mental Health Status

^a^ ¥/CNY represents China Yuan, US$1.00 = ¥ 6.7

Table 2 presents participants’ smoking profiles. Before their partner became pregnant, participants reported an average daily consumption of 6.4 (SD = 6.0) cigarettes. About 53.9% (251/466) of participants attempted to quit smoking before their partner became pregnant, and 33.7% (157/466) chose to smoke outside the home only. Once their partner was pregnant, 30.7% (143/466) had quit smoking for more than 1 month. Among current smokers, 52.9% (171/323) attempted to quit smoking but had relapsed, and 47.1% (152/323) did not attempt to quit after their partner became pregnant. The daily cigarette consumption among current smokers was 6.6 (SD = 9.3). Among current smokers, 36.8% (119/323) reduced their daily cigarette consumption by at least 50% after their partner became pregnant. A total 39.1% (94/323) and 15.5% (50/323) of participants had moderate or high levels of nicotine dependence, respectively. At the time of the survey, 67.5% (218/323) of smokers had no intention to quit within the following 30 days. The mean score of smoking self-efficacy among all participants was 38.7 (SD = 10.4).

**Table 2:** The smoking profiles of the participants (*n* = 466)

	n (%)
**Before partner got pregnant**	
Years of regular smoking, mean (SD)	14.2(6.7)
Daily cigarette consumption, mean (SD)	6.4(6.0)
Quit attempt	
Yes	251(53.9)
No	215(46.1)
Smoke outside the home only	
Yes	157(33.7)
No	309(66.3)
**After partner got pregnant**	
Smoking Status	
Quitter	143(30.7)
Smoker	323(69.3)
Quitted but relapsed^*^	*171(52.9)*
No attempt to quit^*^	*152(47.1)*
Daily cigarette consumption, mean (SD)^*^	6.6(9.3)
The use of other tobacco products	12(3.7)
Reduce cigarette consumption by at least 50%^*^	
Yes	119(36.8)
No	204(63.2)
Heaviness level of Nicotine Dependence scored by FTND*	
Low dependence (0–3)	179(55.4)
Moderate dependence (4, 5)	94(29.1)
High dependence (6–10)	50(15.5)
Readiness to quit within 30 days*	
Yes	105(32.5)
No	218(67.5)
Smoking self-efficacy scored by SEQ-12 (12–60), mean (SD)	38.7(10.4)

Data are n (%) and mean (SD) unless stated otherwise. *FTND* Fagerström Test of Nicotine Dependence, *SEQ-12* Smoking Self-efficacy Questionnaire

^*^Calculation based on participants who were smoker after their partners got pregnant

As shown in Table 3, participants identified an average of 2.06 (SD = 1.43) out of 7 health hazards posed by smoking to smokers themselves, 0.86 (SD = 0.95) of 3 health hazards of SHS to pregnant women, and 2.33 (SD, 1.93) of 7 SHS health hazards to the fetus and newborn. As presented in Table 4, about 66.5% (310/446) of participants agreed that smoking should be prohibited whenever pregnant women and newborns are at home. About 94.2% (439/466) of expectant fathers felt that they should quit smoking for the health of their baby. Approximately 90.8% (423/466) of smoking expectant fathers said that they believed smoking could negatively affect their health; 80.3% (374/466) and 70.8% (330/466) of participants believed that SHS could negatively affect the health of pregnant women, and the fetus and newborn, respectively.

**Table 3:** Knowledge related to the hazard of smoking and secondhand smoke on the health of smoker, pregnant woman, fetus and child (*n* = 466)

	n (%)^a^	95% CI ^b^
**Score of knowledge on the health hazards of smoking to smokers (0–7), mean (SD)**^**c**^	**2.06(1.43)**	**1.93 to 2.19**
Hypertension	132(28.3)	24 to 32%
lung cancer	255(54.7)	50 to 59%
Diabetes	134(28.8)	25 to 33%
Fundus macula	128(27.5)	23 to 32%
Erectile dysfunction	137(29.4)	25 to 34%
Heart disease	101(21.7)	18 to 25%
Sperm malformation	74(15.9)	13 to 19%
**Score of knowledge on the health hazards of SHS to the pregnant women (0–3), mean (SD)**^**c**^	**0.86(0.95)**	**0.77 to 0.94**
Abortion	146(31.3)	27 to 36%
Pregnancy HBP	209(44.8)	40 to 49%
Gestational diabetes	44(9.4)	7 to 12%
**Score of knowledge on the health hazards of SHS to the fetus and newborns (0–7), mean (SD)**^**c**^	**2.33(1.93)**	**2.15 to 2.51**
Fetal death	215(34.5)	42 to 51%
Low birth weight	161(34.5)	30 to 39%
Neural tube deformity	103(22.1)	18 to 26%
Lung dysplasia	64(13.7)	11 to 17%
Newborn death	122(26.2)	22 to 30%
Cough	213(45.7)	41 to 50%
Asthma	208(44.6)	40 to 49%

^a^ The number and proportion of participant correctly identifying the health hazards caused by smoking or SHS on the health of smokers, pregnant women and child

^b^ The 95% confidence interval for the percentage or mean

^c^ The average numbers of diseases in the categories that was correctly identified to be associated with smoking or secondhand smoke by each participants

**Table 4:** Attitude, and perception towards the tobacco use of the participants (*n* = 466)

	n (%)	95% CI ^a^
**Attitude**		
Smoking should be prohibited whenever pregnant women and newborns are at home	310(66.5)	62.2 to 70.8%
I should quit smoking for the health of my baby	439(94.2)	92.1 to 96.3%
**Perception**		
Smoking can negatively affect my health	423(90.8)	88.2 to 93.4%
SHS can negatively affect the health of pregnant	374(80.3)	76.7 to 83.9%
SHS can negatively affect the health of fetus and newborns	330(70.8)	66.7 to 74.9%

^a^ The 95% confidence interval for the percentage

### Factors associated with smoking abstinence among expectant fathers after their partner became pregnant

**Fig. 1:**
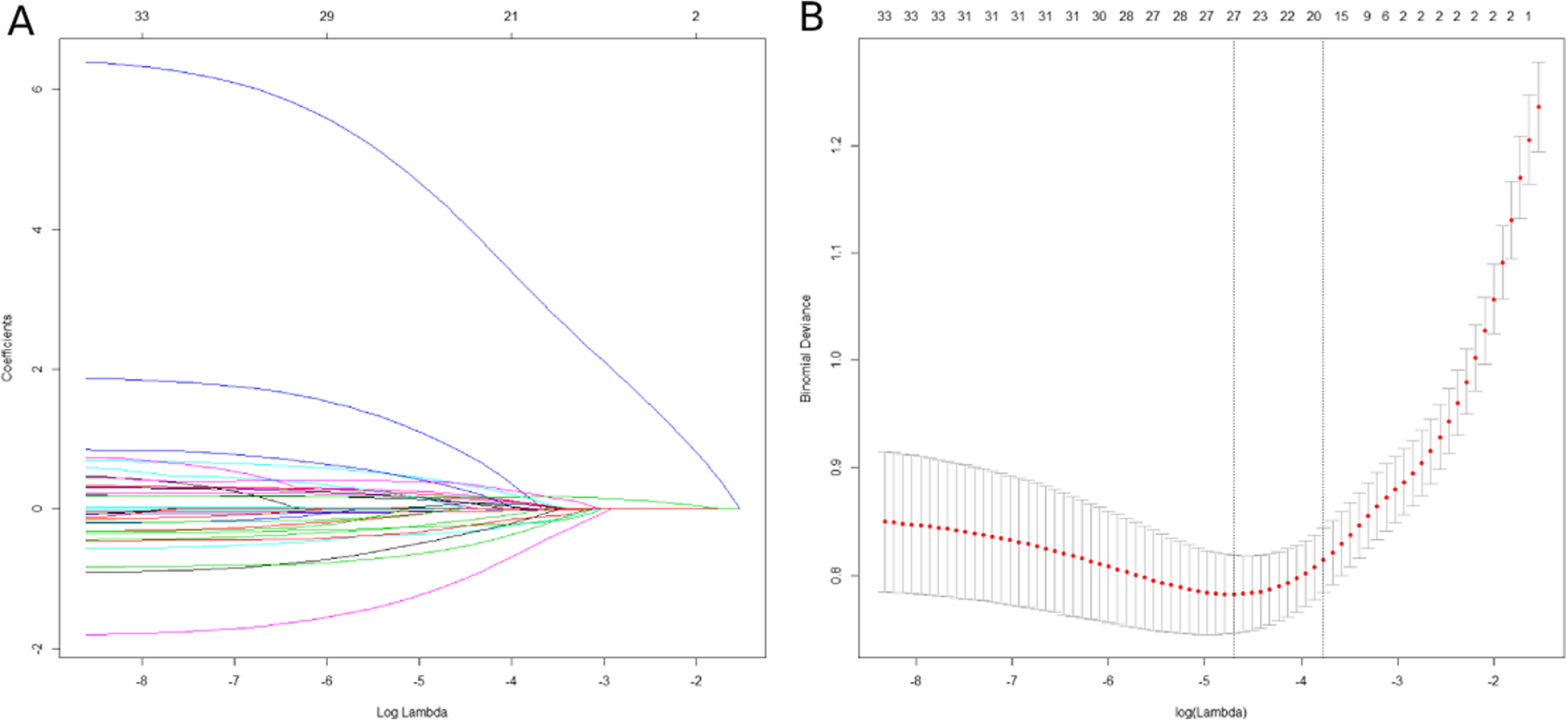
Features selection using the Lasso regression. **a** Lasso regression coefficients. **b** Lasso cross-validation (nfold = 5). LASSO coefficient profiles of the 27 features, including age, employment status, education level, annual family income, monthly alcohol use, regular activity, physical health status, mental health status, stress full events within 30 days, first time expectant fathers, living with smokers, wife is smoker, family APAGAR, prenatal education attendance, receiving smoking cessation advice, daily cigarette consumption before partner got pregnant, quit attempt before partner got pregnant, smoke outside the home only, year of regular tobacco use, knowledge on the health hazards of smoking to smokers, knowledge on the health hazards of SHS to the pregnant women, knowledge on the health hazards of SHS to the fetus and newborns, attitude to ban smoking at home where there are pregnant, attitude to quit smoking for the health of baby, perception towards that smoking can negatively affect my health, perception toward that SHS can negatively affect the health of pregnant, perception toward that SHS can negatively affect the health of fetus and newborns, 19 features with nonzero coefficients was selected

**Fig. 2:**
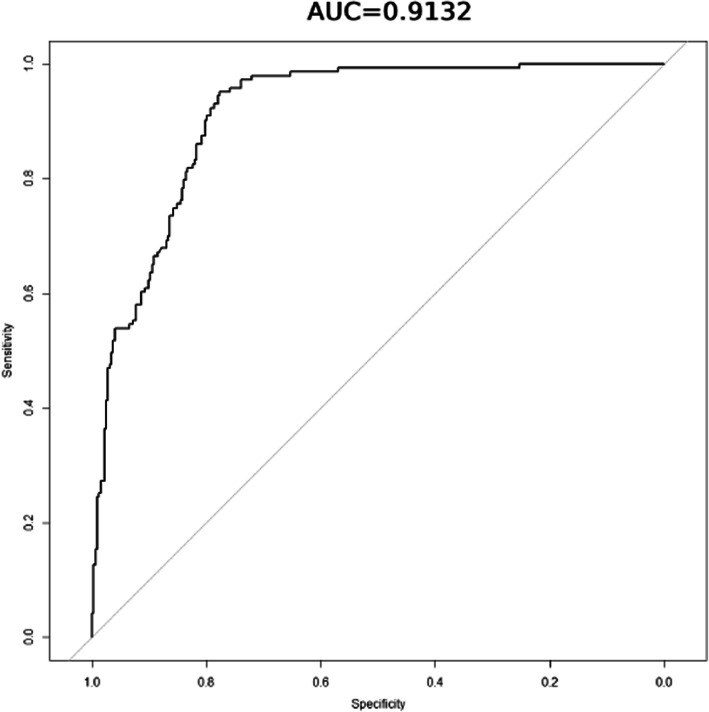
The area under the ROC curve (AUC) of the smoking abstinence predictive nomogram

Using lasso regression, 19 features were selected in the model among 27 independent variables (Fig. 1). The area under the ROC curve (AUC) was 0.913 (Fig. 2) and results of the Hosmer–Lemeshow test (*p* = 0.154) indicated that the selected model was reliable and acceptable for predicting smoking abstinence among smoking expectant fathers.

Table 5 presents the results of multivariable logistic regression with respect to successful smoking abstinence among expectant fathers after their partner became pregnant. Knowledge about the health hazards of smoking to smokers themselves (odds ratio (OR) 1.39; 95% CI 1.24 to 1.58; *p* < 0.001), knowledge about the health hazards of SHS to pregnant women (OR 1.46; 95% CI 1.09 to 1.97; *p* < 0.001) and to the fetus and newborn (OR 1.58; 95% CI 1.25 to 2.03; *p* < 0.001), and being a first-time expectant father (OR 2.08; 95% CI 1.02 to 3.85; *p* = 0.046) were found to be significantly positively associated with smoking abstinence among expectant fathers after their partner became pregnant. Severe dysfunctionality in terms of family support (OR 0.48; 95% CI 0.24 to 0.95; *p* = 0.036) and smoking only outside the home (OR 0.81; 95% CI 0.26 to 0.98; *p* < 0.001) were found to be significantly negatively associated with expectant fathers’ smoking abstinence after their partner became pregnant.

**Table 5:** Multiple logistic regression analyses on predictors of expectant fathers’ abstinence of smoking after partners got pregnant (*n* = 466)

	Adjusted Odds Ration (95% confidence interval)	*p*-value
Age	1.01(0.95 to 1.07)	0.865
Employment status		
Unemployed/Self-employment	1.39(0.65 to 1.95)	0.408
Employed	1	
Education level		
College/university or above	0.74(0.37 to 1.46)	0.388
Middle school or below	1	
Annual Family income (CNY) ^a^		
¥ 50,000–99,999	0.97(0.41 to 2.31)	0.941
¥ 100,000-199,999	0.68(0.30 to 1.54)	0.351
¥ 200,000or above	1.67(0.63 to 4.52)	0.306
¥ 49,999 or below	1	
Monthly regular alcohol use		
Yes	0.71(0.36 to 1.39)	0.319
No	1	
Regular activity at least 1 h/week		
Yes	0.66(0.35 to 1.26)	0.208
No	1	
First time to be expectant father		
Yes	2.08(1.02 to 3.85)	0.046*
No	1	
Family function level, by family APAGR		
Severe dysfunctionality family support (0–3)	0.48(0.24 to 0.95)	0.036*
Moderate dysfunctionality family support (4–7)	0.21(0.04 to 1.00)	0.055
Satisfactory support from family (8–10)	1	
Prenatal education attendance		
Yes	1.49(0.83 to 2.67)	0.180
No		
Daily cigarette consumption before pregnant	0.96(0.91 to 1.00)	0.079
Quit attempt before partner got pregnant		
Yes	0.73(0.71 to 1.09)	0.143
No	1	
Smoke outside the home only		
Yes	0.81(0.26to 0.98)	< 0.001***
No		
Knowledge on the health hazards of smoking to smokers	1.39(1.24 to 1.58)	< 0.001***
Knowledge on the health hazards of SHS to the pregnant women	1.46(1.09 to 1.97)	< 0.001***
Knowledge on the health hazards of SHS to the fetus and newborns	1.58(1.25 to 2.03)	< 0.001***
Attitude to ban smoking at home where there are pregnant	1.36(0.61 to 3.03)	0.448
Perception towards that smoking can negatively affect my health	2.57(0.51 to 12.92)	0.252
Perception toward that SHS can negatively affect the health of pregnant	1.11(0.44 to 2.76)	0.823
Perception toward that SHS can negatively affect the health of fetus and newborns	1.23(0.45 to 3.43)	0.684

^a^ ¥/CNY represents China Yuan, US$1.00 = ¥ 6.7

* *P* < 0.05

*** *P* < 0.001

## Discussion

To our knowledge, this is the first study to examine the smoking behaviors of Chinese expectant fathers and the association with smoking abstinence after their partner becomes pregnant. Our study results showed that more than half (69.3%) of smoking expectant fathers continued to use tobacco after their partner had become pregnant. In addition, among all smoking expectant fathers, 47.1% made no attempt to quit smoking since their partner was pregnant, and 67.5% had no intention to quit within the following 30 days. These findings show that it is crucial for health care professionals to develop and evaluate interventions that can first promote smokers’ intention to quit and then to help them quit smoking step by step [28, 29].

Our study showed that smoking expectant fathers had insufficient knowledge about the relationships between smoking and health hazards of tobacco use to smokers, pregnant women, fetuses, and newborns. Consistent with previous studies [30, 31], the findings of this study provide further support that knowledge among expectant fathers about the health hazards of smoking and SHS to smokers, pregnant women, fetuses, and newborns are a main factor that is associated with smoking abstinence after their partner becomes pregnant. Insufficient smoking-related knowledge, especially regarding hazards to the health of pregnant women, the fetus, and newborns, may result in low motivation and unwillingness to quit smoking among expectant fathers, even after their partner becomes pregnant. It is therefore crucial that health care organizations operationalize greater efforts and resources, so as to implement effective health education and interventions directed toward expectant fathers when they accompany their pregnant partner to an obstetrics and gynecology clinic. Specifically, health care professionals should provide education, to clarify misconceptions among expectant fathers about their smoking habits and reinforce their knowledge about the relationship between smoking and health hazards to their pregnant partner, the fetus, and the newborn.

The results of multivariable regression showed that expectant fathers who only smoked outside the home were less likely to abstain from smoking. A previous study showed that many people misunderstand that if they smoke outside of the home or not in front of others, this will protect non-smokers from the potential harms of SHS [32]. Therefore, smoking expectant fathers might wrongly believe that smoking outside the home is sufficient to prevent their pregnant partner and baby from being exposed to SHS; consequently, these expectant father did not have the intention to quit even after their partner became pregnant. Evidence shows that harmful chemicals on smokers’ clothing and hair, which is called thirdhand smoke, can produce long-term harmful effects to the health of pregnant women and newborns [33]. Thus, information about thirdhand smoke should be provided to smoking expectant fathers in future practice, with the aim to increase their awareness about such long-term health impacts on their pregnant partner and baby, thereby motivating them to abstain from tobacco use.

The results of regression analyses revealed that males who were the first-time to be expectant fathers were more likely to quit smoking after their partner got pregnant. This might be due to the shifts in masculinity associated with impending fatherhood. The transition to fatherhood involved feelings of anticipation about their new paternal role before birth, which increased the eagerness of expectant or new fathers to make positive behavior changes to their smoking behaviors [6]. However, such feeling may be weakened in the subsequent pregnancies [34]. In addition, their previous experience of healthy babies born with paternal smoking during pregnancy and postpartum was at odds with the advice given by health professionals, which may increase their suspicions about the risks of smoking, and consequently weaken their motivation to quit smoking [11, 33, 35]. Health care professionals should therefore pay more attention to men who are not the first-time expectant fathers by assessing their intention to quit smoking so as to develop effective interventions to motivate them to quit smoking.

The results of multivariable regression indicated that severe dysfunctionality in terms of family support was a factor that was negatively associated with smoking abstinence among expectant fathers. There is some evidence that support and encouragement from the partner can motivate expectant fathers to quit smoking and increase the probability of successful abstinence [7]. However, poor family relationships and a lack of the partners’ support might result in less concern among expectant fathers about the relationship between SHS and pregnancy complications or adverse birth outcomes. Consequently, such expectant fathers might have greater reservations about quitting smoking. Thus, apart from offering smoking cessation interventions, it is crucial to refer smokers with severely dysfunctional levels of family support to appropriate organizations for counseling and support, to increase the probability of achieving successful smoking abstinence.

### Limitations of the study

There were several limitations to this study. First, we relied mainly on self-reported smoking status, which is less desirable than biologically-confirmed smoking status. Second, although we encouraged all pregnant women to invite their smoking husband to participate in this study even if they did not accompany them to a prenatal visit, some expectant fathers were reluctant to join the study. Further study may consider to explore whether male partners who attend prenatal visits are more likely to engage with educational interventions than those who do not. Third, the gestation age of the pregnant women at the time of recruitment varied. As this is a cross-sectional study, some fathers may have relapsed and recommenced smoking after data collection. Researchers may consider conducting a longitudinal study in future to monitor the expectant fathers’ smoking status throughout the gestation period of their partners.

### Implications for clinical practice and research

Despite the above limitations, the present findings have important implications for clinical practice and research. Expectant fathers may increase their understanding of their own vulnerability to health risks, emotional responses, and changes in their self-image, which may lead them to be motivated to bring about substantial changes in their health behavior, particularly in terms of taking greater responsibility for their own actions. As the tobacco use rate among expectant fathers in China remains very high, further smoking cessation interventions should be developed, implemented, and evaluated to help this population to quit smoking, especially during the teachable period while their partner is pregnant.

Our results provide useful recommendations for health care professionals in guiding the development of smoking cessation interventions. The findings of this study reveal that knowledge among smoking expectant fathers about the risks of SHS to the health of pregnant women, fetuses, and newborns can serve as powerful motivation for their abstinence from tobacco use. This suggests that educational interventions addressing smoking-related hazards, with a particular focus on maternal and neonatal health, are potentially effective and feasible to motivate smoking expectant fathers to quit smoking.

## Conclusion

The findings of this study indicated that many expectant fathers still smoked during their partner’s pregnancy, which might be attributable to a lack of knowledge and misconceptions about the contexts within which smoking and SHS are hazardous. Innovative educational interventions to deliver information about the hazards of SHS for maternal and neonatal health should be developed and evaluated, to improve the effectiveness and feasibility of health care professionals’ efforts to promote smoking cessation among expectant fathers who smoke.

## Supplementary information

**Additional file 1.**

## Data Availability

The relevant anonymized patient level data, full dataset, technical appendix, and statistical code are available on reasonable request from the corresponding author. Consent for data sharing was not obtained but the presented data are anonymized and risk of identification is low.

## Abbreviations

Lasso regression: Least absolute shrinkage and selection operator regression
OR: Odds Ratio
CI: Confidence interval
SHS: Secondhand smoke
