# Supplementary material for: Association of smoking behavior among Chinese expectant fathers and smoking abstinence after their partner becomes pregnant: a cross-sectional study
Source: BMC Pregnancy Childbirth. 2020 Aug 5;20:449. doi: 10.1186/s12884-020-03148-8 (PMC7405418; doi:10.1186/s12884-020-03148-8)
Supplement: Supplementary file 1 — Additional file 1. [file 12884_2020_3148_MOESM1_ESM.pdf]

Structured Questionnaires

Code：____________________ Date：______________________Hospitals:________________

Instruction:

This questionnaire is used to collect the smoking-related information of smoking expectant fathers. All contents are anonymous and will be kept by a dedicated person to ensure the confidential. Please mark "✓" in the appropriate "☐" ", or fill in the content that suits your situation on "_____".

Part One: Smoking status

A1. Did you ever smoke daily within at least one month in the past year? 1.☐ Yes 0.☐ No

A2. Have you smoked each day in the past month? 1.☐ Yes 0.☐ No

A-1-1. Average ________Cigarettes /day

A-2-0.In the past 30 days: (Please fill in the specific number on the line)
How many days do you smoke from Monday to Friday?
Average _____days/week, Average ___Cigarettes /day
How many days do you smoke from Saturday to Sunday?
Average _____days/week, Average ___Cigarettes /day

A3. When did you firstly attempt to use cigarette?
________ years
(Please fill in the specific number on the line)

A4. How old do you start smoking at least one cigarette a month?________ years

A5. Do you use e-cigarettes in the past month? 1.☐ Yes 0.☐ No (Go to A7）

A6. Does the e-cigarette you used contain nicotine ingredients?
1.☐ yes 0.☐ No 9. ☐ Don’t know

A7. Do you use heated tobacco products in the past month? 1.☐ Yes 0.☐ No

A8. Do you use any other tobacco products in the past month except for traditional cigarettes, electronic cigarettes or heated cigarettes?
0.☐ No (Go to A10） 1.☐ Yes, It’s______（Please fill the category of the tobacco products）

A9. How many such tobacco products have you used in the past month?
A total of _____ times, a total of ______units (please fill in the specific number on the line)

A10. Biochemical validated smoking status

Exhaled CO level		**Reference：≥ 4** ppm

B1. How soon after waking do you smoke your first cigarete?
3. ☐ Within 5 mins 2. ☐ 6-30 mins 1. ☐ 31-60 mins 0. ☐ 60 mins later

B2. Do you find it difficult to refrain from smoking places where it is forbidden? E.g. Church, Library, etc.
1. ☐ Yes 0. ☐ No

B3. Which cigarette would you hate to give up? 1. ☐ The First in the morning 0. ☐ Any other

B4. How many cigarette a day do you smoke?
0. ☐ 10 or less 1. ☐ 11-20 2. ☐ 21-30 3. ☐ 31 or more

B5. Do you smoke more frequently in the morning? 1. ☐ Yes 0. ☐ No

B6. Do you smoke even if you are sick in most of the day? 1. ☐ Yes 0. ☐ No

0To be completed by researcher: Total score: _______
Nicotine Dependency: 1.☐ Mild (0–3) 2.☐ Moderate (4–5) 3.☐ Severe (6 – 10)

Part Two：Quit history

C1. Please recall the situation before your partner got pregnant mentioned in the question below.

C1-1. Did you smoke daily before your partner got pregnant? 1.☐ Yes 0.☐ No

C-1-1-1. Average ________Cigarettes /day

C-1-1-0.In the past 30 days:
How many days do you smoke from Monday to Friday?
Average _____days/week, Average ___Cigarettes /day
How many days do you smoke from Saturday to Sunday?
Average _____days/week, Average ___Cigarettes /day
(Please fill in the specific number on the line)

C1-2. Did you smoke at home before your partner got pregnant?
1. ☐ Yes 0.☐ No

____

（Please fill in the three places where you smoke most frequenty）

C2. How many times have you attempted to quit smoking for more than 24 hours in the past year?
0. ☐ No attempt (Go to D1) 1. ☐ Yes, I attempted________times (Please fill in the specific number on the line)

C3. How long have you sustained to no smoke?________days (Please fill in the specific number on the line)

C4. When was the last time you tried to quit smoking? _________(Please fill in the specific date on the line)

C5. Do you plan to quit smoking now?1. ☐ Yes 0. ☐ No(Pre-contemplation) (Go to D1)

C6. When do you plan to quit smoking? (Single choice)
0. ☐ I have no smoking now (action)
1. ☐ I will quit smoking within 7 days (preparation/contemplation)
2. ☐ I will quit smoking within 1 month (preparation/contemplation)
3. ☐ I will quit smoking within 6 months (contemplation)
4. ☐ I will quit smoking after 6 months (pre-contemplation)
5. ☐ Have not made a decision yet (pre-contemplation)

The exact time
i)___ days after; or
ii)___ months after.

C7. 1. ☐ Decided smoking cessation date ____________________ 0. ☐ Undecided smoking cessation date

C8. The following are some situations in which certain people might be tempted to smoke. Please indicate whether you are sure that you could refrain from smoking in each situation using one of the following answers:

	Not at all sure	Not very sure	More or less sure	Fairly sure	Absolutely sure
1. When I feel nervous.	1. ☐	2. ☐	3. ☐	4. ☐	5. ☐
2. When I feel depressed	1. ☐	2. ☐	3. ☐	4. ☐	5. ☐
3. When I am angry	1. ☐	2. ☐	3. ☐	4. ☐	5. ☐
4. When I feel very anxious	1. ☐	2. ☐	3. ☐	4. ☐	5. ☐
5. When I want to think about a difficult problem	1. ☐	2. ☐	3. ☐	4. ☐	5. ☐
6. When I feel the urge to smoke	1. ☐	2. ☐	3. ☐	4. ☐	5. ☐
7. When having a drink with friends	1. ☐	2. ☐	3. ☐	4. ☐	5. ☐
8. When celebrating something	1. ☐	2. ☐	3. ☐	4. ☐	5. ☐
9. When drinking beer, wine, or other spirits	1. ☐	2. ☐	3. ☐	4. ☐	5. ☐
10. When I am with smokers	1. ☐	2. ☐	3. ☐	4. ☐	5. ☐
11. After a meal	1. ☐	2. ☐	3. ☐	4. ☐	5. ☐
12. When having coffee or tea	1. ☐	2. ☐	3. ☐	4. ☐	5. ☐

C9. Have you used any other smoking cessation service in the past year?

1. ☐ Yes 0. ☐ No（Go to part Three）

C10. What smoking cessation service you have used? (Multiple choices)

	No	Yes	（times used）
1. ☐ Telephone counselling	☐	☐	(_________)
2. ☐ Nicotine replacement treatment	☐	☐	(_________)
3. ☐ Face to Face counselling	☐	☐	(_________)
4. ☐ Group discussion	☐	☐	(_________)
5. ☐ Medication treatment	☐	☐	(_________)
6. ☐ Acupuncture treatment	☐	☐	(_________)
7. ☐ Others：____________________ （Please mark）	☐	☐	(_________)

Part Three: Family situation

D1. How many children do you currently have? _____________ (please fill in specific numbers on the line)

D2. How many smokers you are living with? ____________(please fill in specific numbers on the line)；

Does your partner smoke?

1. ☐ She is a current smoker 2. ☐ She smoked before, but quitted now 0. ☐ She never smoked

D3. Have you attended prenatal education courses before?

0. ☐ No 1. ☐ Yes → Did it include any content related to smoking? 0. ☐ No 1. ☐ Yes

D4. Have you ever received any smoking cessation advice from healthcare professionals?

0. ☐ No 1. ☐ Yes

D5. Please select the option that most suits you based on the following conditions:

Conditions	Almost always	Some of the time	Hardly ever
1. I am satisfied that I can turn to my family for help when something is troubling me.	2. ☐	1. ☐	0. ☐
2. I am satisfied with the way my family talks over things with me and shares problems with me.	2. ☐	1. ☐	0. ☐
3. I am satisfied that my family accepts and supports my wishes to take one new activities or directions.	2. ☐	1. ☐	0. ☐
4. I am satisfied with the wat my family expresses affection and responds to my emotions, such as anger, sorrow, and love.	2. ☐	1. ☐	0. ☐
5. I am satisfied with the way my family and I share time together	2. ☐	1. ☐	0. ☐

Part Five: Health Status

E1. Do you drink? 1. ☐ Yes, the frequency was about: 0. ☐ No, I never drink（Go to E2）

0. ☐ Drink occasionally 1. ☐ 1-2 times per month 2. ☐ 1-2 times per week 4. ☐ More than once a day

E2. Have you done regular physical exercises within the past month? 1. ☐ Yes 0. ☐ No（Go to A3）

What sport do you do? _______________ How often do you do the physical activity? _____mins/week

E3. In general, would you say your health is

5. ☐ Excellent 4. ☐ Very good 3. ☐ Good 2. ☐ Fair 1. ☐ Poor

E4. Within the past year, have you suffered from long-term illness?(Note: Long-term illness means that a certain illness has affected you for a long time or you have been troubled by a certain illness for a long time)

0. ☐ Yes (Go to F5) ☐ No (Go to E6)

E5. If yes, Does the long-term illness now limit your daily activities?

0. ☐ Yes ☐ No

E6. The following items are about activities you might do during a typical day. Does your health now limit you in these activities? If so, how much?

E6-1. Moderate activities, such as moving a table, pushing a vacuum cleaner, bowling, or paying golf.

0.☐ Yes, limited a lot 1. ☐ Yes, limited a little 2. ☐ No, not limited at all

E6-2. Climbing several flights of stairs.

0.☐ Yes, limited a lot 1. ☐ Yes, limited a little 2. ☐ No, not limited at all

E7. During the past week, have you had accomplished less than you would like with your work or other regular daily activities as a result of your physical health?

0.☐ Yes 1. ☐ No

E8. During the past week, have you were limited in the kind of work or other activities as a result of your physical health?

0.☐ Yes 1. ☐ No

E9. During the past week, have you had accomplished less than you would like with your work or other regular daily activities as a result of any emotional problems?

0.☐ Yes 1. ☐ No

E10. During the past week, have you not done work or other activities as carefully as usual as a result of any emotional problems?

0.☐ Yes 1. ☐ No

E11. During the past week, how much did pain interfere with your normal work (including both work outside the hone and housework)?

0. ☐ Not at all 1. ☐ A little bit 2. ☐ Moderately
3. ☐ Quit a bit 4. ☐ Extremely 5. ☐ Not applicable

E12. These questions are about how you feel and how things have been with you during the past week. For each question, please give the one answer that comes closest to the way you have been feeling.

E12-1. How much of the time during the past week have you felt calm and peaceful?

1. ☐ All of the time 2. ☐ Most of the time 3. ☐ A good bit of time
4. ☐ Some of the time 5. ☐ A little of the time 6. ☐ None of the time

E12-2. How much of the time during the past week did you have a lot of energy?

1. ☐ All of the time 2. ☐ Most of the time 3. ☐ A good bit of time
4. ☐ Some of the time 5. ☐ A little of the time 6. ☐ None of the time

E12-2. How much of the time during the past week have you felt downhearted and blue?

1. ☐ All of the time 2. ☐ Most of the time 3. ☐ A good bit of time
4. ☐ Some of the time 5. ☐ A little of the time 6. ☐ None of the time

E12-2.During the past week, how much of the time have your physical health or emotional problems interfered with your social activities (like visiting with friends, relatives, etc.)?

1. ☐ All of the time 2. ☐ Most of the time 3. ☐ A good bit of time
4. ☐ Some of the time 5. ☐ A little of the time 6. ☐ None of the time

Part Five: Attitude and perceptions towards the tobacco use

F1. Do you think the smoking will affect to your health?
1. ☐ Yes, smoking will negatively affect my health
2. ☐ No, smoking has no effect to my health
3. ☐ Not sure

F2. Do you think the SHS will affect to the health of pregnant women
1. ☐ Yes, smoking will negatively affect the health of pregnant women
2. ☐ No, smoking has no effect to the pregnant women
3. ☐ Not sure

F3. Do you think the SHS will affect to the health of fetus and newborns?
1. ☐ Yes, smoking will negatively affect the health of child
2. ☐ No, smoking has no effect to child
3. ☐ Not sure

F4. Do you agree that smoking should be prohibited whenever pregnant women and newborns are at home
1. ☐ Agree 2. ☐ Disagree 3. ☐ Not sure

F5. Do you agree that you should quit smoking for the health of my baby
1. ☐ Agree 2. ☐ Disagree 3. ☐ Not sure

Part Five: Knowledge related to tobacco use

G1. Which diseases below do you think can be induced to smokers by smoking? Please select all the options that you think are suitable (multiple choices)
0. ☐ No one 1. ☐ Hypertension 2. ☐ Lung cancer 3. ☐ Diabetes
4. ☐ Fundus macula 5. ☐ Erectile dysfunction 6. ☐ Fracture 7. ☐ Fall down
8. ☐ Beriberi 9. ☐ Heart disease 10. ☐ Sperm malformation 11. ☐ Azoospermia

G2. Which diseases below do you think can be induced to pregnant women by longterm exposure of secondhand smoke? Please select all the options that you think are suitable (multiple choices)
0. ☐ No one 1. ☐ Vomit 2. ☐ Pregnancy HBP 3. ☐ Frequent urination
4. ☐ Renal failure 5. ☐ Gestational diabetes 6. ☐ Abortion

G3. Which diseases below do you think can be induced to fetus or newborns by longterm exposure of secondhand smoke? Please select all the options that you think are suitable (multiple choices)
0. ☐ No one 1. ☐ Down syndrome 2. ☐ Fetal death 3. ☐ Low birth weight
4. ☐ Hoof kidney 5. ☐ Neural tube deformity 6. ☐ Lung dysplasia 7. ☐ Newborn death
8. ☐ Cough 9. ☐ Congenital heart disease 10. ☐ Asthma 11. ☐ Vitiligo

Part Seven: Demographics information

H1. Age______Years

H2. What is your highest education level?
1. ☐ primary school 2. ☐ Junior Secondary school
3. ☐ Senior secondary school 4. ☐ Degree or above

H2. What's your current employment status：
1. ☐ Employed 2. ☐ Self-employed 3. ☐ Unemployed

H3. How much is your annually family income
0. ☐ 10k or below 1. ☐ 10k-50k 2. ☐ 50k-100k
3. ☐ 100k-200k 4. ☐ 200k or above

——————— Thank you for your cooperation! This is the end of the questionnaire ———————

准爸爸吸烟信息收集问卷

说明：

该问卷是想了解准爸爸吸烟有关的信息，所有内容均为匿名，并且会由专人保管，保证您的信息不会泄露，请在适当的"☐"内打"✓"，或在"_____"上填写符合您情况的内容。

编码：____________ 日期：______________医院：_________________

第一部分：吸烟情况

A11. 您过去一年之内至少有一个月每天都有吸烟吗？ 1.☐ 是 0.☐ 不是

A12. 您过去30天之内每天都有吸烟吗？ 1.☐ 是 0.☐ 不是

A-2-1 平均每天抽_________ 支

A-2-0.过去的 30 天内:（请在横线上填入具体数字）
周一到周五有几天有吸烟？_____天，平均______支/天；
周末有几天有吸烟？_____天，平均_____支/天.

A13. 您在几岁的时候吸第一支烟？________ 岁

A14. 您几岁开始每个月最少吸一支烟呢？ _________岁

A15. 您过去一个月有没有使用电子烟？1.☐ 有 0.☐ 没有(跳到A6）

A16. 您使用的电子烟含不含尼古丁成分？1.☐ 有 0.☐ 没有 9. ☐ 不知道

A17. 您过去一个月有没有使用加热烟？1.☐ 有 0.☐ 没有

A18. 除了传统烟、电子烟或者加热烟，您过去一个月还有没有使用其他烟草产品？

0.☐ 没(跳到D部分） 1.☐ 有，是__________(请填入烟草产品)

A19. 你过去一个月有使用多少这种烟草产品？共_____次，共______个/支（请在横线上填入具体数字）

A20. 生化检测结果

呼吸一氧化碳		**参考值：≥ 4** ppm

B1. 您早晨醒来后多久吸第一支煙？

3.☐ 5 分钟 2.☐ 6-30 分钟内 1.☐ 31-60 分钟内 0.☐ 60 分钟后

B2. 当您身处非吸烟区内，会不会感到难受而忍不住吸烟？ 1. ☐ 会 0. ☐ 不会

B3. 您觉得哪一支烟最难放弃？ 1. ☐早上第一支 0. ☐其它

B4. 您每天抽多少支烟？

0. ☐ 10支或以下 1. ☐ 11-20支 2. ☐ 21-30支 3. ☐ 31支或更多

B5. 当您生病几乎整天卧病在床时还会吸烟吗？ 1. ☐ 会 0. ☐ 不会

B6. 在起床后数小时内，您吸烟次数是否较在其他时间频繁？ 1. ☐ 会 0. ☐ 不会

0To be completed by researcher: Total score: _______
Nicotine Dependency: 1.☐ Mild (0–3) 2.☐ Moderate (4–5) 3.☐ Severe (6 – 10)

第二部分：戒烟史

C1.　请您回忆一下在您太太怀孕之前与以下问题有关的情况。

C1-1.　您太太怀孕之前您每天都有吸烟吗？　1.☐ 是　　0.☐ 不是

C1-1-1 平均每天抽________ 支

C1-1-0.在您太太怀孕之前 (请在横线上填入具体数字）
周一到周五有几天有吸烟？_____天，平均______支/天；
周末有几天有吸烟？_____天，平均_____支/天.

C1-2.　您太太怀孕之前您是否会在家里抽烟?

1.☐ 会　　　0.☐ 不会 → 那您一般在哪里抽烟？ ______________________
(请填入您最经常抽烟的三个场所)

C2.　在过去一年之内，您太太怀孕之前，您是够曾试过真正的戒烟超过24小时以上？
0. ☐ 没有试过　　　1. ☐ 有，试过____________次（请在横线上填入具体数字）

C3.　在过去一年之内，您太太怀孕之后，您是够曾试过真正的戒烟超过24小时以上？
0. ☐ 没有试过　　　1. ☐ 有，试过____________次（请在横线上填入具体数字）

C4.　您最长多长时间没有吸烟？____________天（请在横线上填入具体数字）

C5.　您最近一次尝试戒烟是什么时候？____________（请在横线上填入具体日期）

C6.　您现在有没有打算戒烟？1. ☐ 有　　　0. ☐ 没有(Pre-contemplation) (跳去 D1)

C7.　您打算从什么时候开始戒烟？(单选)
0. ☐ 我现在已经没有吸烟了 (action)
1. ☐ 我会在近7天之内戒烟 (preparation/contemplation)
2. ☐ 我会在近1个月之内戒烟 (preparation/contemplation)
3. ☐ 我会在近6个月之内戒烟 (contemplation)
4. ☐ 我会在6个月之后戒烟 (pre-contemplation)
5. ☐ 还没做好决定 (pre-contemplation)

确切时间：
i)___ 日后 或 ii)___ 月后

C7-1. 1. ☐ 已决定的戒烟日期____________________　　0. ☐ 未决定戒烟日期

C8.　大多数吸烟人士在以下情况都会吸烟，请指出若在同样的情况下，你有多肯定能够在那些吸烟的诱惑下而不吸烟。

	绝对不肯定	不肯定	大概肯定	肯定	绝对肯定
1. 觉得紧张	1. ☐	2. ☐	3. ☐	4. ☐	5. ☐
2. 觉得忧郁不开心	1. ☐	2. ☐	3. ☐	4. ☐	5. ☐
3. 愤怒时	1. ☐	2. ☐	3. ☐	4. ☐	5. ☐
4. 觉得十分焦虑时	1. ☐	2. ☐	3. ☐	4. ☐	5. ☐
5. 想思考难题的时候	1. ☐	2. ☐	3. ☐	4. ☐	5. ☐
6. 当我有吸烟的渴望时	1. ☐	2. ☐	3. ☐	4. ☐	5. ☐
7. 和朋友一起饮酒聊天时	1. ☐	2. ☐	3. ☐	4. ☐	5. ☐
8. 在庆祝轰动的场合	1. ☐	2. ☐	3. ☐	4. ☐	5. ☐
9. 引用啤酒、白酒，或其他含酒精饮料时	1. ☐	2. ☐	3. ☐	4. ☐	5. ☐
10. 身边的人在吸烟	1. ☐	2. ☐	3. ☐	4. ☐	5. ☐
11. 进餐后	1. ☐	2. ☐	3. ☐	4. ☐	5. ☐
12. 喝咖啡或茶的时候	1. ☐	2. ☐	3. ☐	4. ☐	5. ☐

C9. 请问过去一年到目前为止，有没有用过任何其他戒烟辅导服务？

1. ☐ 有　　　　　　　　0. ☐ 没有（跳去第三部分）

C10. 您使用的是什么服务？（可多选）

	没有	有	（次数）
1. ☐ 电话戒烟辅导	☐	☐	(____)
2. ☐ 尼古丁替代治疗	☐	☐	(____)
3. ☐ 面对面辅导	☐	☐	(____)
4. ☐小组讨论戒烟辅导	☐	☐	(____)
5. ☐ 戒烟药物治疗	☐	☐	(____)
6. ☐ 针灸治疗	☐	☐	(____)
7. ☐ 其他：____________________（请注明）	☐	☐	(____)

第三部分·家庭情况

D1. 您目前共有几个孩子？______________(请在横线上填入具体数字）

D2. 和您同住的人之中，有多少人抽烟？_____________人（请在横线上填入具体数字）；

其中您的妻子是否抽烟？1. ☐ 又抽烟　2. ☐ 之前抽已经戒烟了　　0. ☐ 从不抽烟

D3. 您之前是否参加过孕前教育课程？

0. ☐ 没有　　　0. ☐ 有→　是否讲授吸烟与怀（备）孕有关的内容？ 0.☐ 没有　0. ☐ 有

D4. 在过去一年之内您是否收到过专业人士的戒烟建议?

0. ☐ 没有　　　0. ☐ 有

D5. 请根据以下情况选择最符合您的选项

情景	经常这样	有时这样	几乎很少
1. 当我遭遇困难时，可以从家人处得到满意的帮助	2. ☐	1. ☐	0. ☐
2. 我很满意家人与我讨论各种事情以及分担问题的方式	2. ☐	1. ☐	0. ☐
3. 当我希望从事新的活动或发展时，家人都能接受且给予支持	2. ☐	1. ☐	0. ☐
4. 我很满意家人对我表达情感的方式以及对我的情绪（愤怒、悲伤、爱）的反应	2. ☐	1. ☐	0. ☐
5. 我很满意家人与我共度时光的方式	2. ☐	1. ☐	0. ☐

第四部分：健康状况

E1. 您是否饮酒： 1. ☐ 是，饮酒量频率约是什么？　0. ☐ 否（跳到E2）

0. ☐ 偶尔喝一次　　1. ☐ 每月1-2次　　2. ☐ 每周1-2次　　3. ☐ 每天一次以上

E2. 您过一个月内有没有做过运动或者体育锻炼？ 1. ☐ 有　　0. ☐没有（跳到E3）

您做的运动是什么？______________________　　您做运动的频率是多少？_________分钟/星期

E3. 总体来说，您认为您现时的健康状况是

5. ☐ 非常好　4. ☐ 很好　3. ☐好　2. ☐一般　1. ☐差

E4. 在过去一年，您有否患上一些长期疾病？（注：长期疾病是指某一疾病已影响您已有一段很长的时间或您因某一疾病而有一段很长的时间已受到困扰）

0. ☐ 有　☐ 没有**（转至问题E6）**

E5. 如有，您有否因这些疾病而限制了您的日常活动？

0. ☐ 有　☐ 没有

E6. 以下各项是您日常生活中可能进行的活动。以您目前的健康状况，您在进行这些活动时，有没有受到限制？如果有，程度如何？

E6-1. 中等强度的活动，例如搬桌子、打扫或清洁地板，打保龄球，或打太极拳？

0.☐ 有很大限制　1. ☐ 有一点限制　2. ☐没有任何限制

E6-2. 是否影响你步行上楼？

0.☐ 有很大限制　1. ☐ 有一点限制　2. ☐没有任何限制

E7. 在过去一个星期里，您会否因为身体健康的原因，在日常生活或工作中感到力不从心?

0.☐ 会　1. ☐ 不会

E8. 在过去一个星期的工作或日常活动中，您会否因为身体健康的原因而令您的工作或活动受到限制？

0.☐ 会　1. ☐ 不会

E9. 在过去一个星期里，您会否因为情绪方面的原因（比如感到沮丧或焦虑）而令您在工作或日常活动中感到力不从心？

0.☐ 会　1. ☐ 不会

E10. 在过去一个星期的工作或日常活动中，您会否因为情绪方面的原因（比如感到沮丧或者焦虑）而令您的工作或活动受到限制？

0.☐ 会　1. ☐ 不会

E11. 在过去一个星期里，您身体上的疼痛对您的日常工作（包括上班和家务）有多大影响？

0. ☐完全没有影响　1. ☐ 有很少影响　2. ☐ 有一些影响

3. ☐有较大影响　4. ☐有非常大的影响　5. ☐不适用

E12. 以下问题是有关您在过去四个星期里自我感觉及其他的情况。针对每一个问题，请选择一个最接近您感觉的答案。

E12-1. 在过去一个星期里，您有多少时间感到心平气和？

1. ☐ 常常　2. ☐ 大部分时间　3. ☐ 很多时间
4. ☐ 一半　5. ☐ 只有很少时间　6. ☐从来没有

E12-2. 在过去一个星期里，您有多少时间感到精力充足？

1. ☐ 常常　2. ☐ 大部分时间　3. ☐ 很多时间
4. ☐ 一半　5. ☐ 偶尔一次半次　6. ☐从来没有

E12-3. 在过去一个星期里，您有多少时间觉得心情不好、闷闷不乐或沮丧？

1. ☐ 常常　2. ☐ 大部分时间　3. ☐ 很多时间
4. ☐ 一半　5. ☐ 偶尔一次半次　6. ☐从来没有

E12-4. 在过去一个星期里，有多少时间由于您身体健康或情绪问题而妨碍您的社交活动（比如探亲、访友等）？

1. ☐ 常常　2. ☐ 大部分时间　3. ☐ 很多时间
4. ☐ 一半　5. ☐ 偶尔一次半次　6. ☐从来没有

第五部分：吸烟相关态度和认知

F6. 请问您觉得吸烟是否对人的身体健康有害呢？

1. ☐ 有害　2. ☐ 无害　3. ☐ 不确定

F7. 您觉得二手烟会对孕妇造成影响吗？

1. ☐ 会　2. ☐ 不会　3. ☐ 不确定

F8. 您觉得二手烟会对新生儿的健康造成影响吗?

1. ☐ 会　2. ☐ 不会　3. ☐ 不确定

F9. 您是否同意当家中有孕妇和新生儿的时候应该禁止吸烟？

1. ☐ 同意　2. ☐ 不同意　3. ☐ 不确定

F10. 您觉得您会为了孩子的健康应该戒烟吗？

1. ☐ 会　2. ☐ 不会　3. ☐ 不确定

第六部分：吸烟相关知识

G4. 您觉得吸烟会引起一下哪几种疾病呢？请勾选所有您觉得符合的选项（多选）

0. ☐ 没有　1. ☐ 高血压　2. ☐ 肺癌　3. ☐ 糖尿病
4. ☐ 眼底黄斑　5. ☐ 勃起障碍　6. ☐ 骨折　7. ☐ 跌倒
8. ☐ 脚气　9. ☐ 心脏病　10. ☐ 精子畸形　11. ☐ 无精

G5. 您觉得孕妇长期被迫吸食二手烟可能造成哪些影响？请勾选所有您觉得符合的选项（多选）

0. ☐ 没有　1. ☐ 呕吐　2. ☐ 妊娠高血压　3. ☐ 尿频
4. ☐ 肾衰　5. ☐ 妊娠糖尿病　6. ☐ 增加流产率

G6. 您认为长期暴露于二手烟会对孕妇的胎儿或者新生儿造成哪些影响？请勾选所有您觉得符合的选项（多选）

0. ☐ 没有　1. ☐ 唐氏综合征　2. ☐ 胎儿猝死　3. ☐ 出生地体重
4. ☐ 马蹄肾　5. ☐ 神经管畸形　6. ☐ 肺发育不良　7. ☐ 新生儿死亡
8. ☐ 咳嗽　9. ☐ 先天性心脏病　10. ☐ 咳嗽　11. ☐ 白癜风

第七部分：个人资料

H1. 年龄:__________________岁

H2. 您最高的教育程度是：

1. ☐ 小学(含)以下　2. ☐ 初中　3. ☐ 高中　4. ☐ 本科及以上

H3. 您现在的工作状态是：

1. ☐受雇　2. ☐自营　3. ☐无业

H4. 您的家庭人均年收入在以下哪一个范围之内？

1. ☐ 1万以下　1. ☐ 1万-5万　2. ☐ 5万-10万　3. ☐ 10万-20万　4. ☐ 20万以上

————————————多谢您的合作！问卷到此结束————————————
